# Follicular fluid lipidomic profiling reveals potential biomarkers of polycystic ovary syndrome: A pilot study

**DOI:** 10.3389/fendo.2022.960274

**Published:** 2022-09-13

**Authors:** Ying Ding, Yihong Jiang, Mingjiang Zhu, Qinling Zhu, Yaqiong He, Yao Lu, Yuan Wang, Jia Qi, Yifan Feng, Rong Huang, Huiyong Yin, Shengxian Li, Yun Sun

**Affiliations:** ^1^ Center for Reproductive Medicine, Ren Ji Hospital, Shanghai Jiao Tong University School of Medicine, Shanghai, China; ^2^ Shanghai Key Laboratory for Assisted Reproduction and Reproductive Genetics, Shanghai, China; ^3^ Department of Endocrinology and Metabolism, Ren Ji Hospital, Shanghai Jiao Tong University School of Medicine, Shanghai, China; ^4^ CAS Key Laboratory of Nutrition, Metabolism and Food Safety, Shanghai Institute of Nutrition and Health, Shanghai Institutes for Biological Sciences (SIBS), Chinese Academy of Sciences (CAS), Shanghai, China

**Keywords:** polycystic ovary syndrome, follicular fluid, lipidomics, oocyte quality, liquid chromatography-tandem mass spectrometry

## Abstract

**Background:**

Polycystic ovary syndrome (PCOS) is a heterogeneous endocrine disorder associated with multiple metabolic conditions including obesity, insulin resistance, and dyslipidemia. PCOS is the most common cause of anovulatory infertility; however, the molecular diversity of the ovarian follicle microenvironment is not fully understood. This study aimed to investigate the follicular fluid (FF) lipidomic profiles in different phenotypes of PCOS and to explore novel lipid biomarkers.

**Methods:**

A total of 25 women with PCOS and 12 women without PCOS who underwent *in vitro* fertilization and embryo transfer were recruited, and their FF samples were collected for the lipidomic study. Liquid chromatography-tandem mass spectrometry was used to compare the differential abundance of FF lipids between patients with different PCOS phenotypes and controls. Subsequently, correlations between specific lipid concentrations in FF and high-quality embryo rate (HQER) were analyzed to further evaluate the potential interferences of lipid levels with oocyte quality in PCOS. Candidate biomarkers were then compared *via* receiver operating characteristic (ROC) curve analysis.

**Results:**

In total, 19 lipids were identified in ovarian FF. Of these, the concentrations of ceramide (Cer) and free fatty acids (FFA) in FF were significantly increased, whereas those of lysophosphatidylglycerol (LPG) were reduced in women with PCOS compared to controls, especially in obese and insulin-resistant groups. In addition, six subclasses of ceramide, FFA, and LPG were correlated with oocyte quality. Twenty-three lipid subclasses were identified as potential biomarkers of PCOS, and ROC analysis indicated the prognostic value of Cer,36:1;2, FFA C14:1, and LPG,18:0 on HQER in patients with PCOS.

**Conclusions:**

Our study showed the unique lipidomic profiles in FF from women with PCOS. Moreover, it provided metabolic signatures as well as candidate biomarkers that help to better understand the pathogenesis of PCOS.

## Introduction

Polycystic ovary syndrome (PCOS) is a common female endocrine disorder affecting both reproductive and metabolic functions, with a prevalence rate of 5%–10% among women of reproductive age (1–3). PCOS is characterized by the association between clinical and/or biochemical hyperandrogenism and chronic oligo-anovulation and/or ultrasonographic evidence of polycystic ovaries (4). Women with PCOS have a high prevalence of infertility, oligomenorrhoea, androgen excess, obesity, insulin resistance, and dyslipidemia (5–7). Moreover, there is a growing interest in the complications associated with metabolic disturbances in PCOS, which affects the quality of life of numerous women worldwide. However, despite extensive research efforts, understanding the genetic, molecular, and cellular mechanisms underlying the pathophysiology of PCOS is still challenging (8, 9). This highlights the need to further assess the pathogenesis of PCOS and to identify potential biomarkers for prevention of long-term complications *via* appropriate screening, early and accurate diagnosis, and effective intervention.

Previous studies have shown that changes in the follicular microenvironment are correlated with PCOS (10). The search for biomarkers in biologic fluids, including follicular fluid (FF), has emerged as an alternative to invasive methods (11). FF is derived from ovarian antral follicles, which contains important metabolites and serves as a complex microenvironment for follicular development, oocyte maturation, and germ-somatic cell interactions (12). Impaired oocyte quality and outcomes of *in vitro* fertilization (IVF) are related to changes in FF components among patients with PCOS, but the mechanisms have not been fully elucidated yet (13). The metabolomic profiling of FF may have an important impact on oocyte developmental potential and embryo viability. However, whether there are characteristic differences in FF lipid levels between PCOS patients and healthy controls has not been explored. Since intrafollicular communication is critical for normal oocyte development and reproduction, the identification of FF components may provide a better understanding and reveal potential lipid biomarkers of PCOS.

Lipids are essential metabolites that have many crucial cellular functions and can enable a direct readout of cellular metabolic status. Lipidomics is the study of lipidomes using the principles and techniques of analytical chemistry (14, 15). It provides a powerful tool for developing lipid biomarkers to diagnose different diseases. In addition, the discovery of lipid-based biomarkers can be an alternative method for exploring disease states (16). This indicates the potential benefit of clinical lipidomics in the identification and development of disease biomarkers, assessment of signals and metabolic processes, and provision of insights into molecular mechanisms and drug targets. A comprehensive analysis of lipid classes and molecular species has been performed using the separation power of liquid chromatography-mass spectrometry (LC-MS) (17). This post-genomic technology has been applied to rapidly detect global metabolic profiling in biological systems and is a useful strategy for discovering biomarkers, identifying perturbed pathways, diagnosing diseases, and measuring responses to medical treatment (18).

Therefore, the current study aimed to investigate FF lipidomic changes underlying the different phenotypes of PCOS. Here we show that women with PCOS had elevated FF ceramide (Cer) and free fatty acids (FFA) concentrations and decreased FF levels of lysophosphatidylglycerol (LPG), which might affect high-quality embryo rate. Moreover, we demonstrate that Cer,36:1;2, FFA C14:1, and LPG,18:0 could represent candidate lipid biomarkers of embryo quality in PCOS patients. Our findings suggest that altering ovarian lipid metabolism may help improve oocyte quality and early embryo development, which provides a potential intervention on pregnancy outcomes in patients with PCOS.

## Materials and methods

### Participants

Participants were recruited from the Center for Reproductive Medicine, Renji Hospital, Shanghai Jiao Tong University School of Medicine. In total, 25 patients with PCOS and 12 women of similar age without PCOS were included in the study. Patients with PCOS were diagnosed based on the Rotterdam criteria, which exclude other endocrine disorders and require the presence of two or more of the following three signs: oligo- or amenorrhea, clinical and/or biochemical signs of hyperandrogenism, and polycystic ovarian morphology on ultrasound (19). PCOS patients were further divided as follows (1): the obese PCOS (Obese) group (n = 9) with body mass index (BMI) ≥ 25 kg/m^2^ and the lean PCOS (Lean) group (n = 16) with BMI < 25 kg/m^2^; (2) the insulin-resistant PCOS (IR) group (n = 13) with homeostatic model assessment of insulin resistance (HOMA-IR) ≥ 2.5 and the non-insulin-resistant PCOS (NIR) group (n = 12) with HOMA-IR < 2.5 (20); (3) the hyperandrogenism PCOS (HA) group (n = 14) with free androgen index (FAI) ≥ 5 and the non-hyperandrogenism PCOS (NHA) group (n = 11) with FAI < 5 (21). The control group included women with normal ovarian reserve (regular menstrual cycles and AMH concentration of ≥ 2 ng/mL) and normal BMI who presented with infertility caused by fallopian tube disorders or male factors (azoospermia or severe oligo−/asthenoteratozoospermia). All controls had regular menstruation and normal androgen levels, glucose tolerance, and ovarian appearances on ultrasound. Women who received hormonal treatment, insulin sensitizer, anti-hypertensive drugs, or other medications affecting lipid or glucose metabolism within the last 3 months were excluded from the study. This study was approved by the Ethics Committee of Renji Hospital (approval number: 2015030308) and was performed in accordance with the Declaration of Helsinki. A written informed consent was obtained from all participants.

### Clinical measurements and biochemical analyses

BMI was calculated as weight in kilograms divided by the square of height in meters. Fasting blood glucose (FBG) concentration was quantified using the glucose oxidase method (22). The concentrations of follicle-stimulating hormone (FSH), luteinizing hormone (LH), E2, total T, fasting serum insulin (FINS), and sex hormone-binding globulin (SHBG) were assessed using electrochemiluminescence (cobas e 601 module; Roche Diagnostics, Indianapolis, IN, USA). The levels of AMH were evaluated using an enzyme-linked immunosorbent assay kit (Kangrun Biotech, Guangzhou, China) according to the manufacturer’s protocol. Insulin resistance index, as determined by HOMA-IR, was calculated using the following formula: (FBG [in millimoles per milliliter] × FINS [in milliunits per milliliter])/22.5 (23). FAI was calculated as (TT × 100)/SHBG. The participants underwent ultrasound examination using the Voluson E8 System (GE Healthcare, Chicago, IL, USA) between the second and fifth days after menstruation. After the IVF procedure, data on the number of oocytes and oocytes in meiosis II stage (MII), oocyte fertilization rate (defined as the number of oocytes fertilized divided by the number of oocytes inseminated), and pregnancy test results were collected from the participant’s medical record. According to the modified Peter’s scoring system for cleavage-stage embryos (24), the embryos on day 3 after fertilization were divided into the following four grades: grade I - embryos with blastomeres of equal size and fragmentation of < 10%; grade II - embryos with blastomeres of slightly uneven or unequal size with 10%–20% cytoplasmic fragmentation; grade III - embryos with blastomeres of distinctly unequal size with 21%–50% fragmentation; and grade IV - embryos with blastomeres of severe fragmentation with the amount of debris > 50%. Based on these criteria, 6–10 cell and grade I–II embryos on day 3 are considered high-quality embryos, and the rest are regarded as low-quality embryos.

### Sample collection and preparation

Regarding IVF, all patients received controlled ovarian hyperstimulation and medical management according to our established protocols (25). The GnRH antagonist protocol was applied for ovarian stimulation. Each patient received individual doses of gonadotropins, FSH, and/or human menopausal gonadotropin, starting on day 2 or 3 of the menstrual cycle. Recombinant human chorionic gonadotropin (hCG) was administered to the patient to induce final follicular maturation when the dominant follicles reached a diameter of ≥ 18 mm (26, 27). With respect to the standard clinical practice of oocyte retrieval at the Center for Reproductive Medicine, Renji Hospital, all accessible follicles were punctured and aspirated using a 16-gauge needle under ultrasound guidance 35–37 h after the administration of recombinant hCG. Preovulatory ovarian FF samples were collected during oocyte retrieval, and only FF samples with no macroscopic blood contamination were included for further analyses. FF obtained from three dominant follicles with diameters of 18–22 mm was pooled and centrifuged at 1,500 × g for 10 min to remove cellular debris and insoluble particles. The supernatant was then collected and stored at −80°C for further use.

### Lipidomics

Solvents for sample preparation and mass spectrometry (MS) analysis, including methanol, chloroform, and water, were purchased from Burdick and Jackson (Muskegon, MI, USA). Other chemical reagents were purchased from Sigma-Aldrich (St. Louis, MO, USA).

FF lipidomic data were acquired using liquid chromatography with electrospray ionization mass spectrometry (LC-ESI-MS) (28). Details of the experimental protocols including sample preparation and spectroscopy have been described previously (29). Briefly, FF lipids were extracted using a modified methyl tert-butyl ether protocol. Samples were separated on a UPLC HSS T3 column C18 column at 400 μL/min using a gradient comprising mobile phases A and B. Mobile phase A is a mixture of acetonitrile, water, and ammonium acetate. Mobile phase B is a mixture of acetonitrile, isopropanol, and ammonium acetate. MS analysis was performed using a QTRAP 5500 mass spectrometer (SCIEX, Framingham, MA, USA) (30). The mass spectrometer was operated in the negative ion mode *via* multiple reaction monitoring. Analyst 1.6.3 software (SCIEX) was used for data acquisition and processing. FF samples were analyzed in random order, and quality control samples were inserted every eight samples to ensure repeatability. Finally, a total of 581 lipids were quantified.

### Statistical analysis

All data were analyzed using the SPSS 19.0 statistical software (IBM Corp., Armonk, NY, USA). Data distribution was assessed using the normal Quintal plot. Continuous data with a normal distribution were presented as means ± standard deviation (SD). Data with non-normal distributions were analyzed after logarithmic transformation. Comparative analysis of quantitative data was performed using the independent samples *t*-test between the PCOS and control groups. Intergroup comparisons of the measured metabolite intensities were performed by one-way analysis of variance with Bonferroni correction. Categorical variables were expressed as frequency (composition ratio) and were analyzed using the *χ2* test. The statistical relationship between the variables of interest was evaluated using Pearson or Spearman correlation. Receiver operating characteristic (ROC) analysis was performed, and the area under the ROC curve (AUC), 95% confidence interval (CI), and corresponding P values were calculated to establish the predictive value of each parameter in PCOS. A P value of < 0.05 was considered statistically significant.

## Results

### Demographic and clinical characteristics of the study participants

A total of 37 patients were recruited in this study. Twenty-five patients had PCOS based on the Rotterdam criteria. Of the PCOS patients, 16 (64%) had a normal BMI, whereas nine (36%) had a BMI of > 25 kg/m^2^; 13 (52%) patients had insulin resistance classified according to HOMA-IR, and 12 (48%) did not; 11 (44%) patients had no clinical or biochemical evidence of androgen excess, and 14 (56%) patients presented with hyperandrogenism. **Table 1** shows the baseline characteristics and clinical outcomes of patients with different phenotypes of PCOS and controls. Women with and without PCOS did not significantly differ in terms of age (27.92 ± 2.43 vs. 28.16 ± 2.34 years, P = 0.772). A broad spectrum of metabolic and endocrine changes was observed in PCOS patients, including significantly high BMI (23.77 ± 5.18 vs. 20.58 ± 0.91 kg/m^2^, P = 0.006), remarkably elevated serum levels of FINS (12.22 ± 6.59 vs. 5.67 ± 1.18 mIU/L, P < 0.001), basal LH (8.98 ± 4.83 vs. 5.58 ± 2.21 mIU/mL, P = 0.013), AMH (15.14 ± 7.71 vs. 5.81 ± 3.62 ng/mL, P < 0.001), TT (1.69 ± 0.71 vs. 0.69 ± 0.28 nmol/L, P < 0.001), and low levels of basal serum FSH (5.59 ± 1.00 vs. 7.08 ± 1.48 mIU/mL, P = 0.001). As expected, women with PCOS had significantly higher values for HOMA-IR (2.67 ± 1.45 vs. 1.23 ± 0.26, P < 0.001) and FAI (4.78 ± 4.93 vs. 1.29 ± 0.56, P = 0.021) than those without PCOS. There were no statistically significant differences in the FBG and basal E2 levels between the PCOS group and controls (P = 0.914 and 0.112, respectively). Furthermore, subgroup analyses were conducted to further detect the impact of obesity, insulin resistance, and hyperandrogenism in PCOS. Among PCOS patients, obese women showed significantly higher FINS levels (18.00 ± 4.98 vs. 8.97 ± 5.00 mIU/L, P < 0.001), HOMA-IR values (3.95 ± 1.21 vs. 1.95 ± 1.02, P < 0.001), and FAI (10.10 ± 8.12 vs. 3.33 ± 2.77, P < 0.001) than those with a normal BMI; patients with insulin resistance had a significantly higher BMI compared with patients without insulin resistance (26.61 ± 5.60 vs. 20.68 ± 2.08 kg/m^2^, P = 0.001); women with hyperandrogenism had higher BMI (25.69 ± 5.67 vs. 21.32 ± 3.29 kg/m^2^, P = 0.029) and HOMA-IR values (3.18 ± 1.57 vs. 2.03 ± 1.02, P = 0.048) than non-hyperandrogenic women. As compared with controls, the numbers of oocytes retrieved (14.67 ± 6.88 vs. 20.28 ± 8.91, P = 0.036) and MII oocytes (8.67 ± 4.33 vs. 15.08 ± 7.61, P = 0.002) during IVF were significantly higher in PCOS patients. No statistical differences in fertilization rate, high-quality embryo rate (HQER), clinical pregnancy rate, and live birth rate were observed between the groups of patients with PCOS and controls. However, the PCOS IR group had a significantly lower HQER than the control group (0.42 ± 0.27 vs. 0.69 ± 0.23, P = 0.031). Overall, these changes were clinically associated with the phenotypes of PCOS and complemented the metabolomic analysis.

**Table 1:** Demographics and clinical outcomes of women with PCOS and controls.

Parameter	Control(n=12)	PCOS							
		All PCOS(n=25)	P	Classified according to BMI		Classified according to HOMA-IR		Classified according to FAI	
				Lean (n=16)	Obese (n=9)	NIR (n=12)	IR (n=13)	NHA (n=11)	HA (n=14)
Age, years	27.92 ± 2.43	28.16 ± 2.34	0.772	28.25 ± 2.30	28.00 ± 2.55	28.17 ± 2.04	28.15 ± 2.67	27.36 ± 2.54	28.79 ± 2.05
BMI, kg/m^2^	20.58 ± 0.91	23.77 ± 5.18^a^	0.006	20.57 ± 1.90	29.44 ± 4.12^a,b^	20.68 ± 2.08	26.61 ± 5.60^a,c^	21.32 ± 3.29	25.69 ± 5.67^a,d^
FBG, mmol/L	4.92 ± 0.55	4.94 ± 0.56	0.914	4.93 ± 0.40	4.97 ± 0.79	5.03 ± 0.47	4.87 ± 0.68	4.90 ± 0.49	4.98 ± 0.62
FINS, mIU/L	5.67 ± 1.18	12.22 ± 6.59^a^	< 0.001	8.97 ± 5.00	18.00 ± 4.98^a,b^	6.62 ± 2.16	17.40 ± 4.71^a,c^	9.38 ± 4.98	14.46 ± 7.00^a^
HOMA-IR	1.23 ± 0.26	2.67 ± 1.45^a^	< 0.001	1.95 ± 1.02	3.95 ± 1.21^a,b^	1.49 ± 0.46	3.76 ± 1.15^a,c^	2.03 ± 1.02	3.18 ± 1.57^a,d^
Basal FSH, mIU/mL	7.08 ± 1.48	5.59 ± 1.00^a^	0.001	5.65 ± 1.05^a^	5.47 ± 0.94^a^	5.93 ± 1.00	5.27 ± 0.92^a^	5.81 ± 0.99^a^	5.42 ± 1.01^a^
Basal LH, mIU/mL	5.58 ± 2.21	8.98 ± 4.83^a^	0.013	8.45 ± 4.92	9.39 ± 4.95	9.10 ± 5.64	8.87 ± 4.19	7.69 ± 3.21	9.99 ± 5.72
Basal E2, nmol/L	32.15 ± 6.25	41.78 ± 17.22	0.112	38.37 ± 13.04	47.83 ± 22.50	39.17 ± 12.28	44.19 ± 21.01	40.34 ± 16.24	42.91 ± 18.47
AMH, ng/mL	5.81 ± 3.62	15.14 ± 7.71^a^	< 0.001	16.67 ± 9.26^a^	12.41 ± 2.05^a^	17.13 ± 10.49^a^	13.30 ± 3.20^a^	14.61 ± 8.56^a^	15.56 ± 7.27^a^
TT, nmol/L	0.69 ± 0.28	1.69 ± 0.71^a^	< 0.001	1.54 ± 0.63^a^	1.96 ± 0.81^a^	1.59 ± 0.59^a^	1.78 ± 0.83^a^	1.15 ± 0.62	2.12 ± 0.45^a,d^
SHBG, nmol/L	64.88 ± 37.55	51.22 ± 38.63	0.510	59.51 ± 39.32	20.81 ± 14.33	63.68 ± 41.52	28.79 ± 20.61	64.54 ± 38.09	17.91 ± 6.62^a,d^
FAI	1.29 ± 0.56	4.78 ± 4.93^a^	0.021	3.33 ± 2.77	10.10 ± 8.12^a,b^	3.55 ± 2.97	6.99 ± 7.21	2.19 ± 1.11	11.25 ± 4.87^a,d^
No. of oocytes retrieved	14.67 ± 6.88	20.28 ± 8.91^a^	0.036	21.75 ± 9.15	17.67 ± 8.34	22.17 ± 10.61	18.54 ± 7.00	20.18 ± 6.93	20.36 ± 10.48
No. of MII oocyte	8.67 ± 4.33	15.08 ± 7.61^a^	0.002	16.87^a^ ± 7.72	12.00 ± 6.73	16.58 ± 8.74^a^	13.69 ± 6.43	16.73 ± 7.31^a^	13.79 ± 7.86
Nuclear maturation (MII) rate	0.63 ± 0.25	0.75 ± 0.17	0.104	0.78 ± 0.18	0.69 ± 0.15	0.76 ± 0.19	0.74 ± 0.16	0.82 ± 0.16	0.69 ± 0.16
Fertilization rate	0.55 ± 0.16	0.62 ± 0.16	0.194	0.66 ± 0.11	0.55 ± 0.21	0.65 ± 0.12	0.59 ± 0.19	0.63 ± 0.13	0.61 ± 0.18
High-quality embryo rate	0.69 ± 0.23	0.51 ± 0.27	0.054	0.56 ± 0.27	0.42 ± 0.27	0.60 ± 0.25	0.42 ± 0.27^a^	0.55 ± 0.24	0.47 ± 0.29
Clinical pregnancy rate	0.79 (11/14)	0.71 (25/35)	0.843	0.74 (17/23)	0.67 (8/12)	0.71 (12/17)	0.72 (13/18)	0.65 (11/17)	0.78 (14/18)
Live birth rate	0.71 (10/14)	0.63 (22/35)	0.477	0.61 (14/23)	0.67 (8/12)	0.59 (10/17)	0.67 (12/18)	0.53 (9/17)	0.72 (13/18)

Data are presented as mean ± SD. ^a^Compared with the control group, P < 0.05; ^b^Compared with the Lean group, P < 0.05; ^c^Compared with the NIR group, P < 0.05; ^d^Compared with the NHA group, P < 0.05.

AMH, anti-Müllerian hormone; BMI, body mass index; E2, estradiol; FAI, free androgen index; FBG, fasting blood glucose; FINS, fasting serum insulin; FSH, follicle-stimulating hormone; HOMA-IR, homeostatic model assessment of insulin resistance; MII, metaphase II; LH: luteinizing hormone; SHBG, sex hormone-binding globulin; TT, total testosterone.

### Lipidomic changes between healthy controls and patients with PCOS

Lipids play important roles both as basic substrates and as regulators in many metabolic pathways. To compare global variation in the lipidomic profiles of FF between the PCOS and control groups, the lipid content of pooled FF samples was assessed by LC-MS/MS analysis. All samples were analyzed blindly, and nineteen specific lipids were identified in FF (**Supplementary Table 1**). Three lipids were differentially highlighted, and the quantification information are given in **Table 2**. We observed that concentrations of total ceramide and total FFA were elevated in statistically significant manner (P = 0.029 and 0.008, respectively), whereas the levels of LPG were significantly decreased in FF from the patients with PCOS compared with the controls (P < 0.001), thereby indicating the aberrance of lipid metabolism in PCOS.

**Table 2:** Changes in the relative levels of candidate lipids in the FF for discrimination between women with PCOS and without PCOS.

Parameter	Control (n = 12)	PCOS (n = 25)	*P* value	PCOS *vs.* Control
Total Cer	416.24 ± 116.52	520.53 ± 136.39	0.029	↑
Total FFA	75435.12 ± 16657.29	92830.67 ± 17811.83	0.008	↑
LPG	66.27 ± 22.36	19.13 ± 6.87	< 0.001	↓

Data are presented as mean ± SD.

Cer, ceramide; FFA, free fatty acid; LPG, lysophosphatidylglycerol.

**Figure 1:**
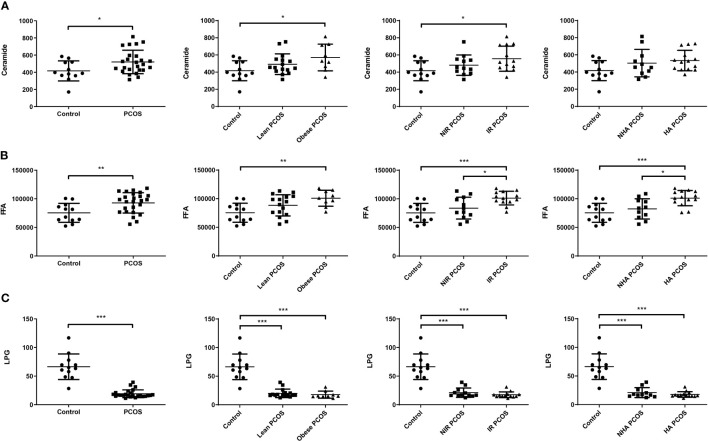
Changes in FF lipids in patients with PCOS (*n* = 25) and healthy controls (*n* = 12). FF ceramide **(A)**, FFA **(B)**, and LPG **(C)** levels in PCOS patients with different phenotypes and controls. Data are expressed as mean ± SD. *P < 0.05, **P < 0.01, ***P < 0.001.

To further understand the potential interaction between biochemical states and lipidomic abnormalities in women with PCOS, we investigated the concentrations of ceramide and LPG in each PCOS subgroup. The concentrations of total ceramide and total FFA showed an increasing trend (P < 0.05) in women with PCOS, while the concentrations of LPG had a decreasing trend (P < 0.001). In particular, ceramide levels in FF were considerably higher in both obese PCOS patients and PCOS patients with insulin resistance than those of controls (P = 0.027 and 0.026, respectively), whereas they did not exhibit statistically significant differences in PCOS women with hyperandrogenism (P = 0.073; **Figure 1A**). As shown in **Figure 1B**, PCOS patients with obesity the had significantly higher FFA levels in FF than controls (P = 0.005). The total FFA levels in PCOS patients with insulin resistance or hyperandrogenism were significantly elevated compared with controls (P < 0.001) and PCOS patients without insulin resistance (P = 0.027) or hyperandrogenism (P = 0.015). Moreover, the concentrations of LPG in each PCOS phenotype were obviously lower than the control group independently of obesity, insulin resistance, and hyperandrogenism (P < 0.001; **Figure 1C**). It is noteworthy that total LPG identified in FF includes LPG with default acyl group and alkyl-LPG. Since no significant difference was found in the alkyl-LPG (LPG O,18:1) level between patients with PCOS and controls, we only analyzed LPG, which represents the default acyl-LPG in this manuscript. Therefore, women with PCOS had an altered FF lipidomic profile in terms of total ceramide, total FFA, and LPG levels.

### Analysis of lipid subclasses and selection of potential biomarkers in the FF of patients with PCOS

To investigate lipidomic differences between the PCOS and control groups and to further identify potential lipid biomarkers, we examined changes in the subclasses of ceramide and LPG in the FF samples. The lipids associated with differences in characteristics among the various groups were identified. As presented in **Table 3**, the FF levels of nine ceramide subclasses (Cer,34:1;2, Cer,36:1;2, Cer,36:2;2, Cer,38:1;2, Cer,38:2;2, Cer,40:0;2, Cer,40:1;2, Cer,40:2;2, and Cer,42:1;2), 11 FFA subclasses (C14:0, C14:1, C16:0, C16:1, C18:1, C18:3, C20:1, C20:4, C20:5, C22:0, and C22:6), and three LPG subclasses (LPG,18:0, LPG,18:1, and LPG,18:2) differed between patients with PCOS and control subjects (P < 0.05). Among the differential lipid subclasses in FF, concentrations of Cer,36:1;2, Cer,36:2;2, Cer,38:1;2, Cer,38:2;2, Cer,40:0;2, FFAs (C14:0, C14:1, C16:0, C16:1, C18:1, C18:3, C20:4, and C20:5) were statistically significantly higher in all of the Obese, IR, and HA groups than in the control group (P < 0.05). In addition, obese PCOS patients had higher FFA C16:0 levels than lean PCOS patients (P = 0.030); the IR group had higher FFAs (C16:0, C18:1, C18:3, and C20:4) concentrations than the NIR group (P < 0.05); the HA group also had significantly higher FF concentrations of FFAs (C16:0, C18:1, and C18:3) than the NHA group (P < 0.05). Furthermore, LPG,18:0 and LPG,18:1 levels were observed to be lower in obese, insulin-resistant, and hyperandrogenic women in the PCOS subgroup than the healthy controls (P < 0.001). The findings suggest that the levels of 23 lipid subclasses were significantly changed in the FF of women with PCOS, and they could potentially serve as the indicator of PCOS.

**Table 3:** FF lipid subclasses in controls and women with PCOS.

Lipid Subclass	Control(n=12)	PCOS							
		All PCOS(n=25)	P	Classified according to BMI		Classified according to HOMA-IR		Classified according to FAI	
				Lean (n=16)	Obese (n=9)	NIR (n=12)	IR (n=13)	NHA (n=11)	HA (n=14)
Cer,34:1;2	54.31 ± 17.49	68.38 ± 20.14^a^	0.046	68.50 ± 21.88	68.16 ± 17.85	67.70 ± 24.47	69.00 ± 16.16	66.58 ± 16.56	69.79 ± 23.08
Cer,36:1;2	7.72 ± 1.58	10.95 ± 3.08^a^	< 0.001	10.39 ± 2.89^a^	11.95 ± 3.31^a^	9.98 ± 2.90^a^	11.85 ± 3.07^a^	9.81 ± 2.84	11.84 ± 3.06^a^
Cer,36:2;2	11.14 ± 4.09	17.89 ± 7.85^a^	0.008	16.14 ± 8.89	21.00 ± 4.45^a^	15.49 ± 10.19	20.10 ± 4.12^a^	15.06 ± 5.76	20.12 ± 8.73^a^
Cer,38:1;2	4.54 ± 1.13	6.27 ± 2.03^a^	0.002	5.98 ± 1.79	6.79 ± 2.42^a^	5.59 ± 1.51	6.90 ± 2.29^a^	5.67 ± 1.95	6.74 ± 2.03^a^
Cer,38:2;2	2.21 ± 0.72	3.04 ± 1.04^a^	0.018	2.78 ± 1.12	3.51 ± 0.73^a^	2.61 ± 1.24	3.44 ± 0.64^a^	2.76 ± 0.93	3.26 ± 1.11^a^
Cer,40:0;2	14.43 ± 5.29	19.92 ± 6.04^a^	0.011	18.43 ± 5.70	22.56 ± 6.02^a^	18.02 ± 5.61	21.67 ± 6.09^a^	18.18 ± 6.01	21.28 ± 5.92^a^
Cer,40:1;2	29.86 ± 9.21	39.03 ± 14.44^a^	0.032	36.06 ± 11.00	44.30 ± 18.70^a^	33.66 ± 8.51	43.98 ± 17.18^a^	37.05 ± 17.83	40.58 ± 11.59
Cer,40:2;2	9.17 ± 3.04	12.35 ± 4.87^a^	0.028	11.33 ± 3.81	14.16 ± 6.18^a^	10.60 ± 3.21	13.97 ± 5.67^a^	11.83 ± 5.23	12.76 ± 4.73
Cer,42:1;2	101.94 ± 37.75	143.00 ± 61.71^a^	0.031	129.90 ± 56.80	166.29 ± 66.51^a^	121.25 ± 42.60	163.08 ± 71.01^a^	131.60 ± 59.70	151.96 ± 63.98
FFA C14:0	225.46 ± 41.17	270.26 ± 46.20^a^	0.007	265.99 ± 51.51	277.85 ± 36.43^a^	250.87 ± 45.70	288.16 ± 40.39^a^	270.00 ± 53.03	270.46 ± 42.16^a^
FFA C14:1	18.52 ± 4.73	29.53 ± 6.33^a^	< 0.001	28.73 ± 7.02^a^	30.96 ± 4.93^a^	27.62 ± 6.45^a^	31.30 ± 5.91^a^	28.19 ± 5.77^a^	30.59 ± 6.75^a^
FFA C16:0	7843.83 ± 1521.67	9715.17 ± 1770.00^a^	0.003	9076.43 ± 1487.33	10850.72 ± 1727.49^a,b^	8657.06 ± 1464.87	10691.89 ± 1464.82^a,c^	8722.47 ± 1484.99	10495.16 ± 1611.98^a,d^
FFA C16:1	894.72 ± 233.15	1265.87 ± 331.47^a^	0.001	1229.92 ± 346.54^a^	1329.77 ± 311.89^a^	1178.28 ± 356.48	1346.73 ± 297.49^a^	1152.35 ± 309.92	1355.06 ± 330.89^a^
FFA C18:1	23446.51 ± 6480.94	30192.11 ± 6002.36^a^	0.004	28138.12 ± 5922.91	33843.65 ± 4343.97^a^	26924.01 ± 6066.24	33208.82 ± 4217.17^a,c^	25444.40 ± 4910.58	33922.46 ± 3729.05^a,d^
FFA C18:3	638.61 ± 211.96	900.21 ± 321.02^a^	0.015	809.68 ± 300.40	1061.14 ± 307.29^a^	721.88 ± 255.10	1064.82 ± 292.05^a,c^	728.31 ± 208.43	1035.27 ± 334.93^a,d^
FFA C20:1	731.96 ± 224.56	907.02 ± 233.85^a^	0.038	888.34 ± 271.64	940.24 ± 154.18	869.15 ± 299.21	941.98 ± 156.69	852.25 ± 300.46	950.06 ± 164.28
FFA C20:4	2542.89 ± 673.50	3731.62 ± 1233.04^a^	0.004	3516.01 ± 1234.55	4114.93 ± 1202.17^a^	3129.24 ± 1033.11	4287.67 ± 1168.16^a,c^	3243.78 ± 1216.73	4114.93 ± 1143.89^a^
FFA C20:5	69.32 ± 30.24	137.82 ± 64.95^a^	0.001	130.50 ± 61.31^a^	150.84 ± 72.89^a^	128.74 ± 65.89^a^	146.20 ± 65.57^a^	115.94 ± 61.33	155.01 ± 64.59^a^
FFA C22:0	33.75 ± 9.48	38.60 ± 6.67^a^	0.040	38.07 ± 6.53	39.53 ± 7.21	37.60 ± 6.87	39.51 ± 6.63	36.90 ± 6.28	39.93 ± 6.89
FFA C22:6	3531.41 ± 1114.99	5216.42 ± 1848.96^a^	0.006	5319.68 ± 2184.56^a^	5032.86 ± 1117.44	5020.88 ± 2471.28	5396.93 ± 1079.58^a^	4876.29 ± 2269.82	5483.67 ± 1474.26^a^
LPG,18:0	55.82 ± 20.68	14.52 ± 5.70^a^	< 0.001	15.42 ± 5.91^a^	12.92 ± 5.24^a^	16.54 ± 6.41^a^	12.65 ± 4.41^a^	15.98 ± 7.06^a^	13.37 ± 4.28^a^
LPG,18:1	5.38 ± 2.91	1.00 ± 0.65^a^	< 0.001	1.04 ± 0.55^a^	0.92 ± 0.83^a^	1.01 ± 0.55^a^	0.99 ± 0.75^a^	1.10 ± 0.63^a^	0.92 ± 0.67^a^
LPG,18:2	5.07 ± 1.49	3.62 ± 1.29^a^	0.004	3.48 ± 1.45^a^	3.86 ± 0.98	3.48 ± 1.68^a^	3.75 ± 0.85	3.85 ± 1.55	3.44 ± 1.07^a^

Data are presented as mean ± SD. ^a^Compared with the control group, P < 0.05; ^b^Compared with the Lean group, P < 0.05; ^c^Compared with the NIR group, P < 0.05; ^d^Compared with the NHA group, P < 0.05.

Cer, ceramide; FFA, free fatty acid; LPG, lysophosphatidylglycerol.

### Correlations between FF lipids and oocyte quality

**Figure 2:**
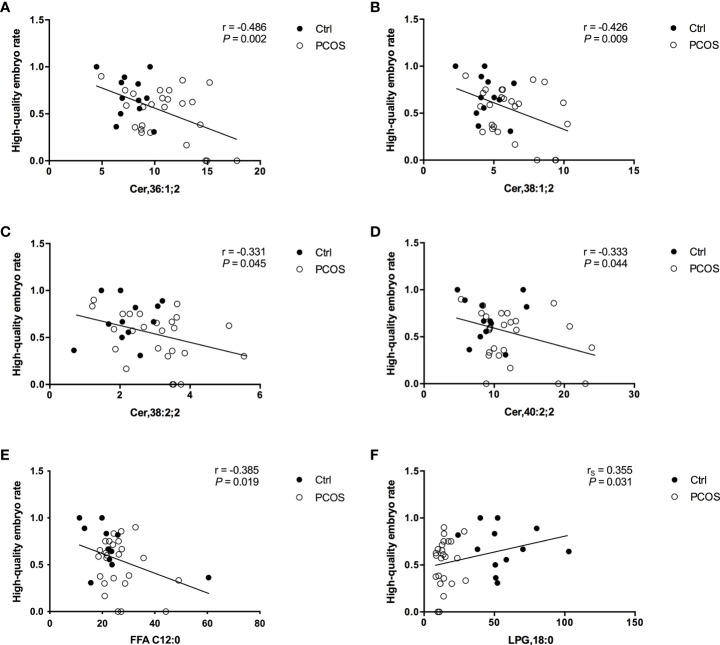
Correlation between specific FF lipids and embryo quality in patients with (white dots; *n* = 25) and without (black dots; *n* = 12) PCOS. Regression analysis between HQER and Cer,36:1;2 **(A)**, Cer,38:1;2 **(B)**, Cer,38:2;2 **(C)**, Cer,40:0;2 **(D)**, FFA C12:0 **(E)**, and LPG,18:0 **(F)** respectively was performed. The lines indicate the fitted regression curves.

To further evaluate the potential interferences between FF lipid levels and oocyte quality in PCOS, the correlations between the levels of specific lipids and HQER were assessed. Six lipid subclasses were found to be highly correlated with oocyte quality using the Pearson or Spearman correlation tests in women with and without PCOS (**Figure 2**). Further linear regression analysis confirmed that the FF concentrations of Cer,36:1;2 (rs = –0.486, P = 0.002), Cer,38:1;2 (rs = –0.426, P = 0.009), Cer,38:2;2 (rs = –0.331, P = 0.045), Cer,40:0;2 (rs = –0.333, P = 0.044), and FFA C12:0 (rs = –0.385, P = 0.019) were significantly negatively associated with HQER (**Figures 2A–E**). Conversely, the levels of LPG,18:0 in FF showed a significantly positive correlation with HQER (rs = 0.355, P = 0.031; **Figure 2F**). We also found that the FF levels of differential lipids were closely correlated with the endocrine-metabolic parameters (**Supplementary Table 2**).

### Identification of potential lipids responsible for distinguishing between women with and without PCOS

**Figure 3:**
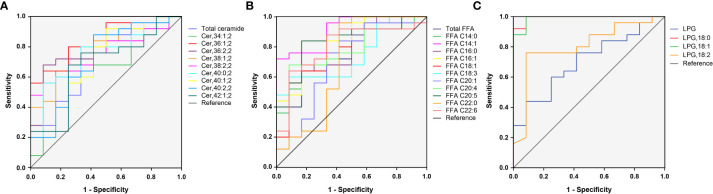
ROC curves for 23 selected lipid subclasses for differentiating women with PCOS from normal healthy individuals. The AUC value of Cer,36:1;2, FFA C14:1, and LPG,18:0 was larger than that of the total ceramide **(A)**, total FFA **(B)**, and LPG **(C)**, respectively.

In total, 23 potential lipidomic biomarkers were identified by MS analysis. When evaluated alone Cer,36:1;2 yielded an AUC of 0.837, which was stronger than for total ceramide concentrations (0.713; **Figure 3A**). The AUC value of FFA C14:1 was higher than that of total FFA concentrations (0.913 vs. 0.763; **Figure 3B**). Among LPG, LPG,18:0 had the highest AUC (0.993; **Figure 3C**), indicating a strong impact of LPG activity.

Since some lipids have significant changes between PCOS subgroups, such as NIR/IR and NHA/HA, we selected four candidate lipid subclasses according to **Table 3**, and then ROC curve analysis was performed for PCOS subgroups. The AUC value of FFA C16:0 (0.827) was higher than that of the other lipid subclasses for distinguishing between PCOS women with and without insulin resistance (**Supplementary Table 3**), and FFA C18:1 yielded the highest AUC (0.903) for distinguishing between NHA and HA subgroups (**Supplementary Table 4**), indicating that these lipids could be used as subtype indicators of PCOS.

## Discussion

Ovarian FF constitutes the microenvironment for follicle development and oocyte maturation. The FF comprises secretions from the granulosa cells (GCs), theca cells, and oocytes within or surrounding the follicle. The composition of FF has attracted considerable interest, and several metabolomic studies, including those on carbohydrate, amino acid, and lipid metabolism, have been conducted (31–35). However, research on sphingolipid metabolism, especially ceramide and its subclass in the FF of PCOS patients with different phenotypes is limited. In the previous study, we found that the content of ceramide in the serum of patients with PCOS was significantly higher than that of BMI-matched controls, which is similar to those reported by Li et al. (36, 37). We also found that the two subclasses Cer (OH_N16:0/N18:0) and Cer (N22:0) may act as lipid biomarkers for predicting PCOS and may play an important role in the pathogenesis of PCOS (36). Therefore, the current study aimed to investigate the lipidomic profiles of FF in PCOS patients with different phenotypes and their association with IVF outcomes.

Ceramide is an important lipid signaling molecule regulated by tumor necrosis factor-α, interferon-γ, Fas ligands, interleukin-1, and nerve growth factor. Ceramide is closely correlated with insulin resistance, metabolic inflammation, and non-alcoholic fatty liver disease (38–40). For example, elevated ceramide levels in the adipose tissue have been observed in obese patients and exacerbated metabolic disorders by inducing adipose tissue inflammation. Ceramide inhibits the protein phosphatase 2A (PP2A) or protein kinase C ζ/Akt signaling pathway, leading to insulin resistance (41, 42). We observed that patients with PCOS had a higher concentration of FF ceramides than controls. Further analysis showed that the ceramide levels in the Obese and IR groups were significantly higher than that of the control group, whereas no difference was seen between the HA and control groups, indicating that obesity or insulin resistance is significantly associated with high ceramide concentrations, rather than testosterone concentrations.

Moreover, ceramide and its metabolites are also involved in the regulation of cell differentiation, proliferation, and apoptosis (43). Eliyahu et al. (44) revealed that acid ceramidase (AC), a sphingolipid hydrolase that hydrolyzes ceramide into sphingosine, is highly expressed in FF, cumulus cells, and oocytes. Recombinant AC could enhance the survival rates and quality of oocytes and embryos grown *in vitro*, as well as embryo development *in vivo* after implantation, while conditional knockout of AC in mice showed elevated ceramide level in the ovary with a correspondingly significant decrease in fertility. These data indicate that high ceramide levels could impair fertility (45). Consistently, we also observed that Cer,36:1;2, Cer,38:1;2, Cer,38:2;2, and Cer,40:2;2 were negatively associated with HQER.

Oocyte maturation is dependent on the activation of the PI3K/Akt signaling pathway (46). Elevated ceramide concentration inhibits the Akt signaling pathway (40, 47), which may impair oocyte maturation. In addition, accumulating evidence suggests that intracellular free Ca^2+^ levels play an important role in the regulation of oocyte maturation and early embryonic development (48–50). The endoplasmic reticulum (ER) may be a vital mediator of Ca^2+^ homeostasis *via* the inositol (1, 4, 5)-trisphosphate receptor (IP3-R) and sarco/ER Ca^2+^-ATPase (SERCA) channels (51, 52). Ginzburg et al. (53) found that the inhibition of SERCA by saccharides alone is extremely weak, and the inhibitory effects increase significantly when the saccharides was attached to a ceramide backbone. Ceramide increases the concentration of IP3 in a dose-dependent manner *via* the activation of the Gq/11 and phospholipase C (PLC) signal pathway. IP3 triggers calcium release from the ER by activating IP3-R in the oocyte of *Xenopus laevis* (54). Calcium oscillations triggered by ceramide *via* the Gq/11-PLC-IP3 pathway may be involved in the regulation of calcium homeostasis during oocyte maturation and follicle development.

Patients with PCOS had higher FF FFA levels than controls. The total FFA levels were significantly increased in the Obese, IR, and HA groups compared with the control group. Furthermore, the IR and HA groups had significantly higher total FFA levels than the NIR and NHA groups respectively, thus suggesting that insulin resistance and hyperandrogenemia increase lipolysis and release more FFA into the circulation (55, 56). Further analysis revealed that the levels of 11 FFA subclasses (C14:0, C14:1, C16:0, C16:1, C18:1, C18:3, C20:1, C20:4, C20:5, C22:0, and C22:6) were increased in PCOS group. In addition, the IR and HA groups had higher C16:0, C18:1, and C18:3 levels than the NIR and NHA groups, while only the IR group had a higher C20:4 level than the NIR group. Palmitic acid (PA, C16:0), the most abundant saturated fatty acid in the circulation, is the main fuel for β oxidation and produces energy. It also causes lipid toxicity, which in turn leads to metabolic inflammation and insulin resistance. PA remarkedly suppresses GC survival in a time- and dose-dependent manner leading to reproductive abnormalities (57). Arachidonic acid (AA, C20:4) is one of the most abundant omega-6 polyunsaturated fatty acids in the blood and is a precursor to inflammatory mediators such as prostaglandins (PGE2, PGD2, PGI2, PGF2a, and PGJ2). Prostaglandins produced by the cyclooxygenase metabolic pathway are involved in different reproductive processes, such as follicle development, oocyte maturation, ovulation, and embryo implantation. Elevated AA levels in FF may produce excessive prostaglandins, leading to local inflammation, which in turn affects the follicle development and ovulation (58–60).

Herein we report that levels of three LPG subclasses (LPG,18:0, LPG,18:1, and LPG,18:2) were significantly lower in PCOS patients than in control subjects. Phosphatidylglycerol (PG) was first discovered in *Lactobacillus arabinoses* by Benson and Maruo in 1958, and it is widely found in animal plants and almost all bacteria. In animals, PG is mainly stored in the plasma membrane. With very few researches on the physiological and pathological role of PG and LPG, neither clinical nor basic studies on the relation between LPG and PCOS have been performed. We found a significant decline in FF LPG of patients with PCOS and no difference between the IR and NIR, HA, and NHA groups, suggesting that PCOS patients have decreased levels of LPG in FF with a unique mechanism. Additionally, the level of LPG O,18:1 was not associated with HQER in women with and without PCOS, and its ROC AUC was only 0.540. The effect of LPG on follicle development and oocyte maturation needs to be further assessed.

This study has several limitations. First, the relatively small sample size affected statistical efficiency. Second, FF samples were collected from patients who received gonadotropin stimulation and hence may not represent their natural state. Third, the composition of FF was highly variable and was associated with the developmental stage of follicles. Therefore, our findings could only reflect changes in the lipid composition of FF just before ovulation.

## Conclusions

The present study found that patients with PCOS had elevated FF ceramide and FFA concentrations and decreased FF levels of LPG, especially in Obese and IR groups. High ceramide and FFA levels and low LPG levels might impair follicle development and oocyte maturation, which then affect HQER. Moreover, we showed that Cer,36:1;2, FFA C14:1, and LPG,18:0 could represent useful lipid biomarkers of embryo quality in PCOS patients. Thus, altering ovarian ceramide, FFA, and LPG synthesis or metabolism may help improve oocyte function and early embryo development, which provides a potential intervention for the treatment of PCOS.

## Data availability statement

The original contributions presented in the study are included in the article/**Supplementary Material**. Further inquiries can be directed to the corresponding authors.

## Ethics statement

The studies involving human participants were reviewed and approved by the Ethics Committee of Renji Hospital. The patients/participants provided their written informed consent to participate in this study.

## Author contributions

YD, SL, and YS designed the study. YL collected clinical information. QZ and YH performed biochemical analysis. YW and JQ collected patient samples. MZ and HY performed MS analysis. YJ, YF, and RH analyzed the data. YD and YJ wrote the manuscript. SL and YS revised the manuscript. All authors contributed to the article and approved the submitted version.

## Funding

This work was supported by National Key R&D Program of China (grant number: 2019YFA0802604), the National Natural Science Foundation of China (grant numbers: 82130046, 82001517), Shanghai Leading Talent Program, Innovative Research Team of High-level Local Universities in Shanghai (grant numbers: SHSMU-ZLCX20210200, SSMU-ZLCX20180401), Clinical Research Plan of SHDC (SHDC2020CR1046B), and Shanghai Municipal Education Commission-Gaofeng Clinical Medicine Grant Support (grant number: 20161413).

## Acknowledgments

We would like to thank Shanghai Institutes for Biological Sciences, Chinese Academy of Sciences for expert technical assistance.

## Conflict of interest

The authors declare that the research was conducted in the absence of any commercial or financial relationships that could be construed as a potential conflict of interest.

## Publisher’s note

All claims expressed in this article are solely those of the authors and do not necessarily represent those of their affiliated organizations, or those of the publisher, the editors and the reviewers. Any product that may be evaluated in this article, or claim that may be made by its manufacturer, is not guaranteed or endorsed by the publisher.

## References

[1] Diamanti-Kandarakis E Dunaif A. Insulin resistance and the polycystic ovary syndrome revisited: An update on mechanisms and implications. Endocr. Rev (2012) 33(6):981–1030. doi: 10.1210/er.2011-1034 23065822 PMC5393155

[2] Azziz R Carmina E Chen Z Dunaif A Laven JS Legro RS. Polycystic ovary syndrome. Nat Rev Dis primers. (2016) 2:16057. doi: 10.1038/nrdp.2016.57 27510637

[3] Jayasena CN Franks S. The management of patients with polycystic ovary syndrome. Nat Rev Endocrinol. (2014) 10(10):624–36. doi: 10.1038/nrendo.2014.102 25022814

[4] Azziz R Carmina E Dewailly D Diamanti-Kandarakis E Escobar-Morreale HF Futterweit W. Positions statement: Criteria for defining polycystic ovary syndrome as a predominantly hyperandrogenic syndrome: An androgen excess society guideline. J Clin Endocrinol Metab (2006) 91(11):4237–45. doi: 10.1210/jc.2006-0178 16940456

[5] Teede H Deeks A Moran L. Polycystic ovary syndrome: a complex condition with psychological, reproductive and metabolic manifestations that impacts on health across the lifespan. BMC Med (2010) 8:41. doi: 10.1186/1741-7015-8-41 20591140 PMC2909929

[6] He Y Lu Y Zhu Q Wang Y Lindheim SR Qi J. Influence of metabolic syndrome on female fertility and *in vitro* fertilization outcomes in PCOS women. Am J obstet. gynecol. (2019) 221(2):138.e1–.e12. doi: 10.1016/j.ajog.2019.03.011 30910544

[7] Balen AH Morley LC Misso M Franks S Legro RS Wijeyaratne CN. The management of anovulatory infertility in women with polycystic ovary syndrome: An analysis of the evidence to support the development of global WHO guidance. Hum Reprod update. (2016) 22(6):687–708. doi: 10.1093/humupd/dmw025 27511809

[8] Morris S Grover S Sabin MA. What does a diagnostic label of 'polycystic ovary syndrome' really mean in adolescence? A review of current practice recommendations. Clin Obes (2016) 6(1):1–18. doi: 10.1111/cob.12123 26568133

[9] Simoni M Tempfer CB Destenaves B Fauser BC. Functional genetic polymorphisms and female reproductive disorders: Part I: Polycystic ovary syndrome and ovarian response. Hum Reprod update. (2008) 14(5):459–84. doi: 10.1093/humupd/dmn024 PMC2515090 18603647

[10] Dai G Lu G. Different protein expression patterns associated with polycystic ovary syndrome in human follicular fluid during controlled ovarian hyperstimulation. Reprod Fertil. Dev (2012) 24(7):893–904. doi: 10.1071/RD11201 22935150

[11] Zamah AM Hassis ME Albertolle ME Williams KE. Proteomic analysis of human follicular fluid from fertile women. Clin Proteomics. (2015) 12(1):5. doi: 10.1186/s12014-015-9077-6 25838815 PMC4357057

[12] Da Broi MG Giorgi VSI Wang F Keefe DL Albertini D Navarro PA. Influence of follicular fluid and cumulus cells on oocyte quality: Clinical implications. J Assist Reprod Genet (2018) 35(5):735–51. doi: 10.1007/s10815-018-1143-3 PMC5984887 29497954

[13] Wang B Li J Yang Q Zhang F Hao M Guo Y. Decreased levels of sRAGE in follicular fluid from patients with PCOS. Reprod (Cambridge England). (2017) 153(3):285–92. doi: 10.1530/REP-16-0359 27965400

[14] Han X Gross RW. Global analyses of cellular lipidomes directly from crude extracts of biological samples by ESI mass spectrometry: A bridge to lipidomics. J Lipid Res (2003) 44(6):1071–9. doi: 10.1194/jlr.R300004-JLR200 12671038

[15] Lee SH Williams MV DuBois RN Blair IA. Targeted lipidomics using electron capture atmospheric pressure chemical ionization mass spectrometry. Rapid Commun Mass Spectrom. (2003) 17(19):2168–76. doi: 10.1002/rcm.1170 14515314

[16] Zhang L Han X Wang X. Is the clinical lipidomics a potential goldmine? Cell Biol Toxicol (2018) 34(6):421–3. doi: 10.1007/s10565-018-9441-1 PMC6208904 30032454

[17] Cajka T Fiehn O. Comprehensive analysis of lipids in biological systems by liquid chromatography-mass spectrometry. Trends Analyt. Chem (2014) 61:192–206. doi: 10.1016/j.trac.2014.04.017 PMC4187118 25309011

[18] Yuan M Breitkopf SB Yang X Asara JM. A positive/negative ion-switching, targeted mass spectrometry-based metabolomics platform for bodily fluids, cells, and fresh and fixed tissue. Nat Protoc (2012) 7(5):872–81. doi: 10.1038/nprot.2012.024 PMC3685491 22498707

[19] Rotterdam ESHRE/ASRM-Sponsored PCOS Consensus Workshop Group. Revised 2003 consensus on diagnostic criteria and long-term health risks related to polycystic ovary syndrome. Fertil. steril. (2004) 81(1):19–25. doi: 10.1016/j.fertnstert.2003.10.004 14711538

[20] Fukushima M Taniguchi A Sakai M Doi K Nagasaka S Tanaka H. Homeostasis model assessment as a clinical index of insulin resistance. Comparison minim. Model anal. Diabetes Care (1999) 22(11):1911–2. doi: 10.2337/diacare.22.11.1911 10546034

[21] Ibanez L Ong KK Lopez-Bermejo A Dunger DB de Zegher F. Hyperinsulinaemic androgen excess in adolescent girls. Nat Rev Endocrinol. (2014) 10(8):499–508. doi: 10.1038/nrendo.2014.58 24776733

[22] McMillin JM. Clinical methods: the history, physical, and laboratory examinations. Butterworth: Butterworth Publishers (1990) 21250045

[23] Matthews DR Hosker JP Rudenski AS Naylor BA Treacher DF Turner RC. Homeostasis model assessment: Insulin resistance and beta-cell function from fasting plasma glucose and insulin concentrations in man. Diabetologia. (1985) 28(7):412–9. doi: 10.1007/BF00280883 3899825

[24] Brinsden PR. A textbook of in vitro fertilization and assisted reproduction: The Bourn Hall guide to clinical and laboratory practice. Boca Raton, Florida: CRC press (1999).

[25] Ding Y Zhu Q He Y Lu Y Wang Y Qi J. Induction of autophagy by beclin-1 in granulosa cells contributes to follicular progesterone elevation in ovarian endometriosis. Trans Res J Lab Clin Med (2021) 227:15–29. doi: 10.1016/j.trsl.2020.06.013 32640290

[26] Ludwig M Doody KJ Doody KM. Use of recombinant human chorionic gonadotropin in ovulation induction. Fertil. steril. (2003) 79(5):1051–9. doi: 10.1016/S0015-0282(03)00173-0 12738494

[27] Lu Y Niu Y Wang Y He Y Ding Y Lu X. Optimal candidates to do fresh embryo transfer in those using oral contraceptive pretreatment in IVF cycles. Front Physiol (2021) 12:576917. doi: 10.3389/fphys.2021.576917 33776782 PMC7991902

[28] Merrill AH Jr. Sullards MC Allegood JC Kelly S Wang E. Sphingolipidomics: high-throughput, structure-specific, and quantitative analysis of sphingolipids by liquid chromatography tandem mass spectrometry. Methods (San Diego Calif). (2005) 36(2):207–24. doi: 10.1016/j.ymeth.2005.01.009 15894491

[29] Yun H Sun L Wu Q Zong G Qi Q Li H. Associations among circulating sphingolipids, β-cell function, and risk of developing type 2 diabetes: A population-based cohort study in China. PloS Med (2020) 17(12):e1003451. doi: 10.1371/journal.pmed.1003451 33296380 PMC7725305

[30] Chew WS Seow WL Chong JR Lai MKP Torta F Wenk MR. Sphingolipidomics analysis of large clinical cohorts. part 1: Technical notes and practical considerations. Biochem Biophys Res Commun (2018) 504(3):596–601. doi: 10.1016/j.bbrc.2018.04.076 29654754

[31] Jove M Pradas I Naudi A Rovira-Llopis S Banuls C Rocha M. Lipidomics reveals altered biosynthetic pathways of glycerophospholipids and cell signaling as biomarkers of the polycystic ovary syndrome. Oncotarget (2018) 9(4):4522–36. doi: 10.18632/oncotarget.23393 PMC5796992 29435121

[32] Haoula Z Ravipati S Stekel DJ Ortori CA Hodgman C Daykin C. Lipidomic analysis of plasma samples from women with polycystic ovary syndrome. Metabolomics. (2015) 11(3):657–66. doi: 10.1007/s11306-014-0726-y PMC4419155 25972770

[33] Zhao Y Fu L Li R Wang LN Yang Y Liu NN. Metabolic profiles characterizing different phenotypes of polycystic ovary syndrome: Plasma metabolomics analysis. BMC Med (2012) 10:153. doi: 10.1186/1741-7015-10-153 23198915 PMC3599233

[34] Zhao X Xu F Qi B Hao S Li Y Li Y. Serum metabolomics study of polycystic ovary syndrome based on liquid chromatography-mass spectrometry. J Proteome Res (2014) 13(2):1101–11. doi: 10.1021/pr401130w 24428203

[35] Jia C Xu H Xu Y Xu Y Shi Q. Serum metabolomics analysis of patients with polycystic ovary syndrome by mass spectrometry. Mol Reprod Dev (2019) 86(3):292–7. doi: 10.1002/mrd.23104 30624822

[36] Jiang Y Qi J Xue X Huang R Zheng J Liu W. Ceramide subclasses identified as novel lipid biomarker elevated in women with polycystic ovary syndrome: a pilot study employing shotgun lipidomics. Gynecol. Endocrinol (2020) 36(6):508–12. doi: 10.1080/09513590.2019.1698026 31793360

[37] Li J Xie LM Song JL Yau LF Mi JN Zhang CR. Alterations of sphingolipid metabolism in different types of polycystic ovary syndrome. Sci Rep (2019) 9(1):3204. doi: 10.1038/s41598-018-35685-w 30824725 PMC6397209

[38] Chaurasia B Tippetts TS Mayoral Monibas R Liu J Li Y Wang L. Targeting a ceramide double bond improves insulin resistance and hepatic steatosis. Science (2019) 365(6451):386–92. doi: 10.1126/science.aav3722 PMC6787918 31273070

[39] Samuel VT Shulman GI. Nonalcoholic fatty liver disease, insulin resistance, and ceramides. New Engl J Med (2019) 381(19):1866–9. doi: 10.1056/NEJMcibr1910023 31693811

[40] Bikman BT Summers SA. Ceramides as modulators of cellular and whole-body metabolism. J Clin Invest. (2011) 121(11):4222–30. doi: 10.1172/JCI57144 PMC3204836 22045572

[41] Holland WL Summers SA. Sphingolipids, insulin resistance, and metabolic disease: new insights from *in vivo* manipulation of sphingolipid metabolism. Endocr. Rev (2008) 29(4):381–402. doi: 10.1210/er.2007-0025 18451260 PMC2528849

[42] Holland WL Brozinick JT Wang LP Hawkins ED Sargent KM Liu Y. Inhibition of ceramide synthesis ameliorates glucocorticoid-, saturated-fat-, and obesity-induced insulin resistance. Cell Metab (2007) 5(3):167–79. doi: 10.1016/j.cmet.2007.01.002 17339025

[43] Chalfant CE Spiegel S. Sphingosine 1-phosphate and ceramide 1-phosphate: expanding roles in cell signaling. J Cell Sci (2005) 118 20:4605–12. doi: 10.1242/jcs.02637 16219683

[44] Eliyahu E Shtraizent N Martinuzzi K Barritt J He X Wei H. Acid ceramidase improves the quality of oocytes and embryos and the outcome of *in vitro* fertilization. FASEB J (2010) 24(4):1229–38. doi: 10.1096/fj.09-145508 PMC3231947 20007509

[45] Eliyahu E Shtraizent N Shalgi R Schuchman EH. Construction of conditional acid ceramidase knockout mice and *in vivo* effects on oocyte development and fertility. Cell Physiol Biochem (2012) 30(3):735–48. doi: 10.1159/000341453 PMC3741991 22854249

[46] Hsueh AJ Kawamura K Cheng Y Fauser BC. Intraovarian control of early folliculogenesis. Endocr. Rev (2015) 36(1):1–24. doi: 10.1210/er.2014-1020 25202833 PMC4309737

[47] Stoica BA Movsesyan VA PMt L Faden AI. Ceramide-induced neuronal apoptosis is associated with dephosphorylation of akt GSK-3beta, and induction of the mitochondrial-dependent intrinsic caspase pathway. Mol Cell Neurosci (2003) 22(3):365–82. doi: 10.1016/S1044-7431(02)00028-3 12691738

[48] Homa ST Carroll J Swann K. The role of calcium in mammalian oocyte maturation and egg activation. Hum Reprod (Oxford England). (1993) 8(8):1274–81. doi: 10.1093/oxfordjournals.humrep.a138240 8408526

[49] Sousa M Barros A Silva J Tesarik J. Developmental changes in calcium content of ultrastructurally distinct subcellular compartments of preimplantation human embryos. Mol Hum Reprod (1997) 3(2):83–90. doi: 10.1093/molehr/3.2.83 9239713

[50] Whitaker M. Calcium at fertilization and in early development. Physiol Rev (2006) 86(1):25–88. doi: 10.1152/physrev.00023.2005 16371595 PMC3299562

[51] Zhang DX Li XP Sun SC Shen XH Cui XS Kim NH. Involvement of ER-calreticulin-Ca2+ signaling in the regulation of porcine oocyte meiotic maturation and maternal gene expression. Mol Reprod Dev (2010) 77(5):462–71. doi: 10.1002/mrd.21166 20222029

[52] Johnson S Michalak M Opas M Eggleton P. The ins and outs of calreticulin: From the ER lumen to the extracellular space. Trends Cell Biol (2001) 11(3):122–9. doi: 10.1016/S0962-8924(01)01926-2 11306273

[53] Ginzburg L Li SC Li YT Futerman AH. An exposed carboxyl group on sialic acid is essential for gangliosides to inhibit calcium uptake *via* the sarco/endoplasmic reticulum Ca2+-ATPase: Relevance to gangliosidoses. J Neurochem (2008) 104(1):140–6. doi: 10.1111/j.1471-4159.2007.04983.x 18173730

[54] Kobrinsky E Spielman AI Rosenzweig S Marks AR. Ceramide triggers intracellular calcium release *via* the IP(3) receptor in xenopus laevis oocytes. Am J Physiol (1999) 277(4):C665–72. doi: 10.1152/ajpcell.1999.277.4.C665 10516096

[55] Arner P. Effects of testosterone on fat cell lipolysis. species differences and possible role in polycystic ovarian syndrome. Biochimie. (2005) 87(1):39–43. doi: 10.1016/j.biochi.2004.11.012 15733735

[56] Ek I Arner P Bergqvist A Carlstrom K Wahrenberg H. Impaired adipocyte lipolysis in nonobese women with the polycystic ovary syndrome: A possible link to insulin resistance? J Clin Endocrinol Metab (1997) 82(4):1147–53. doi: 10.1210/jc.82.4.1147 9100587

[57] Mu YM Yanase T Nishi Y Tanaka A Saito M Jin CH. Saturated FFAs, palmitic acid and stearic acid, induce apoptosis in human granulosa cells. Endocrinology (2001) 142(8):3590–7. doi: 10.1210/endo.142.8.8293 11459807

[58] Dennis EA Norris PC. Eicosanoid storm in infection and inflammation. Nat Rev Immunol (2015) 15(8):511–23. doi: 10.1038/nri3859 PMC4606863 26139350

[59] Segi E Haraguchi K Sugimoto Y Tsuji M Tsunekawa H Tamba S. Expression of messenger RNA for prostaglandin e receptor subtypes EP4/EP2 and cyclooxygenase isozymes in mouse periovulatory follicles and oviducts during superovulation. Biol Reprod (2003) 68(3):804–11. doi: 10.1095/biolreprod.102.003590 12604629

[60] Arbab F Goldsby J Matijevic-Aleksic N Huang G Ruan KH Huang JC. Prostacyclin is an autocrine regulator in the contraction of oviductal smooth muscle. Hum Reprod (Oxford England). (2002) 17(12):3053–9. doi: 10.1093/humrep/17.12.3053 12456602

