# Supplementary material for: Follicular fluid lipidomic profiling reveals potential biomarkers of polycystic ovary syndrome: A pilot study
Source: Front Endocrinol (Lausanne). 2022 Sep 13;13:960274. doi: 10.3389/fendo.2022.960274 (PMC9513192; doi:10.3389/fendo.2022.960274)
Supplement: Supplementary file 1 [file Table_1.docx]

**Supplementary Table 1.** The relative levels of FF lipids between women with PCOS and without PCOS

| **Parameter** | **Control (n = 12)** | **PCOS (n = 25)** | ***P* value** |
| --- | --- | --- | --- |
| CE | 39481.61 ± 4988.15 | 42512.50 ± 7230.28 | 0.200 |
| Cer | 416.24 ± 116.52 | 520.53 ± 136.39 | 0.029 |
| DAG | 104859.90 ± 26429.49 | 127075.61 ± 35603.03 | 0.063 |
| FFA | 75435.12 ± 16657.29 | 92830.67 ± 17811.83 | 0.008 |
| LPA | 968.63 ± 884.64 | 1465.72 ± 1148.54 | 0.191 |
| LPC | 440659.28 ± 39668.21 | 450243.96 ± 73271.59 | 0.675 |
| LPE | 307413.80 ± 31111.59 | 292151.38 ± 50968.70 | 0.348 |
| LPG | 280.82 ± 73.22 | 218.47 ± 85.00 | 0.036 |
| LPI | 858.89 ± 356.42 | 803.84 ± 390.77 | 0.538 |
| LPS | 251.19 ± 146.21 | 284.42 ± 126.25 | 0.119 |
| MAG | 14173.08 ± 1858.60 | 14604.59 ± 2391.14 | 0.586 |
| PA | 571122.16 ± 494069.16 | 688654.81 ± 421840.45 | 0.207 |
| PC | 392424.43 ± 38593.62 | 408897.16 ± 36652.20 | 0.217 |
| PE | 69990.23 ± 11124.88 | 75662.17 ± 25659.16 | 0.471 |
| PG | 12347.34 ± 2715.00 | 13243.55 ± 3972.06 | 0.486 |
| PI | 39785.72 ± 15131.37 | 47652.40 ± 19311.53 | 0.145 |
| PS | 15912.47 ± 6463.11 | 17328.94 ± 8564.27 | 0.243 |
| SM | 33926.50 ± 3187.74 | 34600.77 ± 4822.39 | 0.663 |
| TAG | 235770.75 ± 44495.11 | 271218.91 ± 54890.07 | 0.060 |

Data are presented as mean ± SD.

Abbreviations: CE, cholesterol ester; Cer, ceramide; DAG, diacylglycerol; FFA, free fatty acid; LPA, lysophosphatidic acid; LPC, lysophosphatidylcholine; LPE, lysophosphatidylethanolamine; LPG, lysophosphatidylglycerol; LPI, Lysophosphatidylinositol; LPS, lipopolysaccharides; MAG, monoacylglycerol; PA, phosphatidic acid; PC, phosphatidylcholine; PE, phosphatidylethanolamine; PG, phosphatidylglycerol; PI, phosphatidylinositol; PS, phosphatidylserine; SM, sphingomyelin; TAG, triacylglycerol.
