# Supplementary material for: Follicular fluid lipidomic profiling reveals potential biomarkers of polycystic ovary syndrome: A pilot study
Source: Front Endocrinol (Lausanne). 2022 Sep 13;13:960274. doi: 10.3389/fendo.2022.960274 (PMC9513192; doi:10.3389/fendo.2022.960274)
Supplement: Supplementary file 2 [file Table_2.docx]

**Supplementary Table 2.** The correlations between the differential lipids in FF and endocrine-metabolic parameters in patients with and without PCOS

| **Parameter** | **BMI, kg/m^2^** | | **FBG, mmol/L** | | **FINS, mIU/L** | | **HOMA-IR** | | **Basal FSH, mIU/mL** | | **Basal LH, mIU/mL** | | **Basal E2, nmol/L** | | **AMH, ng/mL** | | **TT, nmol/L** | | **SHBG, nmol/L** | | **FAI** | |
| --- | --- | --- | --- | --- | --- | --- | --- | --- | --- | --- | --- | --- | --- | --- | --- | --- | --- | --- | --- | --- | --- | --- |
|  | **r** | **P** | **r** | **P** | **r** | **P** | **r** | **P** | **r** | **P** | **r** | **P** | **r** | **P** | **r** | **P** | **r** | **P** | **r** | **P** | **r** | **P** |
| **Cer,34:1;2** | -0.030 | 0.860 | -0.232 | 0.166 | 0.135 | 0.424 | 0.084 | 0.620 | -0.109 | 0.520 | 0.292 | 0.079 | -0.016 | 0.924 | 0.078 | 0.647 | 0.253 | 0.131 | 0.132 | 0.591 | -0.180 | 0.462 |
| **Cer,36:1;2** | 0.303 | 0.069 | -0.189 | 0.264 | 0.410^*^ | 0.012 | 0.378^*^ | 0.021 | -0.231 | 0.169 | 0.333^*^ | 0.044 | 0.045 | 0.791 | 0.269 | 0.108 | 0.414^*^ | 0.011 | -0.196 | 0.422 | 0.185 | 0.449 |
| **Cer,36:2;2** | 0.667^*^ | <0.001 | -0.076 | 0.653 | 0.520^*^ | 0.001 | 0.523^*^ | 0.001 | -0.372^*^ | 0.023 | -0.089 | 0.599 | 0.057 | 0.739 | 0.379^*^ | 0.021 | 0.362^*^ | 0.028 | -0.578^*^ | 0.010 | 0.577^*^ | 0.010 |
| **Cer,38:1;2** | 0.148 | 0.382 | -0.377^*^ | 0.021 | 0.416^*^ | 0.010 | 0.345^*^ | 0.037 | -0.276 | 0.098 | 0.187 | 0.269 | -0.111 | 0.511 | 0.286 | 0.086 | 0.230 | 0.170 | -0.297 | 0.217 | 0.263 | 0.277 |
| **Cer,38:2;2** | 0.611^*^ | <0.001 | -0.101 | 0.552 | 0.539^*^ | 0.001 | 0.534^*^ | 0.001 | -0.317 | 0.056 | -0.116 | 0.494 | 0.111 | 0.513 | 0.266 | 0.112 | 0.305 | 0.066 | -0.534^*^ | 0.018 | 0.529^*^ | 0.020 |
| **Cer,40:0;2** | 0.376^*^ | <0.001 | -0.225 | 0.181 | 0.384^*^ | 0.019 | 0.353^*^ | 0.032 | -0.290 | 0.082 | 0.087 | 0.609 | 0.033 | 0.846 | 0.285 | 0.087 | 0.205 | 0.224 | -0.292 | 0.225 | 0.321 | 0.180 |
| **Cer,40:1;2** | 0.259 | 0.122 | -0.322 | 0.052 | 0.401^*^ | 0.014 | 0.339^*^ | 0.040 | -0.347^*^ | 0.036 | 0.014 | 0.935 | -0.088 | 0.604 | 0.275 | 0.100 | 0.153 | 0.367 | -0.199 | 0.415 | 0.149 | 0.543 |
| **Cer,40:2;2** | 0.255 | 0.128 | -0.320 | 0.054 | 0.447^*^ | 0.006 | 0.387^*^ | 0.018 | -0.361^*^ | 0.028 | 0.071 | 0.678 | -0.036 | 0.832 | 0.299 | 0.072 | 0.202 | 0.229 | -0.159 | 0.516 | 0.069 | 0.779 |
| **Cer,42:1;2** | 0.274 | 0.101 | -0.342^*^ | 0.038 | 0.377^*^ | 0.022 | 0.319 | 0.054 | -0.307 | 0.065 | -0.021 | 0.904 | -0.095 | 0.578 | 0.313 | 0.059 | 0.122 | 0.470 | -0.295 | 0.220 | 0.278 | 0.250 |
| **FFA C14:0** | 0.414^*^ | 0.011 | -0.254 | 0.129 | 0.578^*^ | <0.001 | 0.552^*^ | <0.001 | -0.365^*^ | 0.026 | 0.156 | 0.357 | -0.154 | 0.364 | 0.285 | 0.087 | 0.144 | 0.396 | -0.541^*^ | 0.017 | 0.336 | 0.159 |
| **FFA C14:1** | 0.352^*^ | 0.033 | -0.249 | 0.137 | 0.609^*^ | <0.001 | 0.596^*^ | <0.001 | -0.400^*^ | 0.014 | 0.359^*^ | 0.029 | 0.160 | 0.344 | 0.441^*^ | 0.006 | 0.505^*^ | 0.001 | -0.323 | 0.178 | 0.378 | 0.111 |
| **FFA C16:0** | 0.495^*^ | 0.002 | -0.273 | 0.102 | 0.588^*^ | <0.001 | 0.552^*^ | <0.001 | -0.330^*^ | 0.046 | 0.120 | 0.480 | 0.014 | 0.936 | 0.350^*^ | 0.034 | 0.277 | 0.097 | -0.695^*^ | 0.001 | 0.594^*^ | 0.007 |
| **FFA C16:1** | 0.372^*^ | 0.023 | -0.191 | 0.257 | 0.513^*^ | 0.001 | 0.499^*^ | 0.002 | -0.319 | 0.054 | 0.276 | 0.099 | 0.013 | 0.937 | 0.336^*^ | 0.042 | 0.370^*^ | 0.024 | -0.630^*^ | 0.004 | 0.580^*^ | 0.009 |
| **FFA C18:1** | 0.463^*^ | 0.004 | -0.201 | 0.233 | 0.565^*^ | <0.001 | 0.540^*^ | 0.001 | -0.345^*^ | 0.036 | 0.210 | 0.213 | 0.063 | 0.712 | 0.336^*^ | 0.026 | 0.396^*^ | 0.015 | -0.709^*^ | 0.001 | 0.581^*^ | 0.009 |
| **FFA C18:3** | 0.394^*^ | 0.016 | -0.169 | 0.317 | 0.623^*^ | <0.001 | 0.611^*^ | <0.001 | -0.348^*^ | 0.035 | 0.221 | 0.189 | 0.155 | 0.361 | 0.246 | 0.143 | 0.374^*^ | 0.023 | -0.772^*^ | <0.001 | 0.707^*^ | 0.001 |
| **FFA C20:1** | 0.278 | 0.096 | -0.226 | 0.179 | 0.321 | 0.052 | 0.279 | 0.094 | -0.223 | 0.184 | 0.132 | 0.436 | 0.012 | 0.943 | 0.234 | 0.164 | 0.162 | 0.338 | -0.314 | 0.191 | 0.157 | 0.521 |
| **FFA C20:4** | 0.417^*^ | 0.010 | -0.108 | 0.524 | 0.597^*^ | <0.001 | 0.596^*^ | <0.001 | -0.361^*^ | 0.028 | 0.095 | 0.575 | -0.132 | 0.438 | 0.331^*^ | 0.045 | 0.293 | 0.079 | -0.698^*^ | 0.001 | 0.512^*^ | 0.025 |
| **FFA C20:5** | 0.484^*^ | 0.002 | -0.210 | 0.211 | 0.384^*^ | 0.019 | 0.353^*^ | 0.032 | -0.448^*^ | 0.005 | -0.094 | 0.582 | -0.256 | 0.126 | 0.226 | 0.178 | 0.276 | 0.098 | -0.627^*^ | 0.004 | 0.593^*^ | 0.007 |
| **FFA C22:0** | 0.140 | 0.408 | -0.264 | 0.115 | 0.177 | 0.295 | 0.130 | 0.444 | -0.111 | 0.512 | 0.050 | 0.767 | -0.144 | 0.394 | 0.206 | 0.220 | 0.038 | 0.822 | -0.197 | 0.418 | 0.166 | 0.498 |
| **FFA C22:6** | 0.233 | 0.166 | -0.395^*^ | 0.015 | 0.394^*^ | 0.016 | 0.322 | 0.052 | -0.364^*^ | 0.027 | -0.003 | 0.985 | -0.168 | 0.319 | 0.305 | 0.066 | 0.109 | 0.521 | -0.461^*^ | 0.047 | 0.335 | 0.161 |
| **LPG,18:0** | -0.303 | 0.068 | 0.204 | 0.226 | -0.529^*^ | <0.001 | -0.557^*^ | <0.001 | 0.465^*^ | 0.004 | -0.267 | 0.111 | -0.313 | 0.059 | -0.594^*^ | <0.001 | -0.611^*^ | <0.001 | 0.344 | 0.149 | -0.491^*^ | 0.033 |
| **LPG,18:1** | -0.354^*^ | 0.031 | -0.564^*^ | <0.001 | -0.427^*^ | 0.008 | -0.420^*^ | 0.010 | 0.473^*^ | 0.003 | -0.204 | 0.226 | -0.127 | 0.452 | -0.645^*^ | <0.001 | -0.564^*^ | <0.001 | 0.250 | 0.301 | -0.382 | 0.107 |
| **LPG,18:2** | 0.184 | 0.275 | -0.574^*^ | <0.001 | -0.156 | 0.357 | -0.162 | 0.339 | 0.260 | 0.120 | -0.200 | 0.235 | -0.228 | 0.174 | -0.450^*^ | 0.005 | -0.574^*^ | <0.001 | -0.014 | 0.955 | -0.209 | 0.391 |

Data are presented as mean ± SD. ^*^P < 0.05.

Abbreviations: AMH, anti-Müllerian hormone; BMI, body mass index; E2, estradiol; FAI, free androgen index; FBG, fasting blood glucose; FINS, fasting serum insulin; FSH, follicle-stimulating hormone; HOMA-IR, homeostatic model assessment of insulin resistance; LH: luteinizing hormone; SHBG, sex hormone-binding globulin; TT, total testosterone.
