# Supplementary material for: Follicular fluid lipidomic profiling reveals potential biomarkers of polycystic ovary syndrome: A pilot study
Source: Front Endocrinol (Lausanne). 2022 Sep 13;13:960274. doi: 10.3389/fendo.2022.960274 (PMC9513192; doi:10.3389/fendo.2022.960274)
Supplement: Supplementary file 3 [file Table_3.docx]

**Supplementary Table** **3.** Results of ROC analyses for distinguishing between PCOS women with and without IR

| **Lipid** | **AUC (95% CI)** | ***P* value** |
| --- | --- | --- |
| FFA C16:0 | 0.827 (0.658, 0.995) | 0.006 |
| FFA C18:1 | 0.795 (0.620, 0.969) | 0.012 |
| FFA C18:3 | 0.821 (0.650, 0.991) | 0.007 |
| FFA C20:4 | 0.776 (0.588, 0.964) | 0.019 |
