# Supplementary material for: Follicular fluid lipidomic profiling reveals potential biomarkers of polycystic ovary syndrome: A pilot study
Source: Front Endocrinol (Lausanne). 2022 Sep 13;13:960274. doi: 10.3389/fendo.2022.960274 (PMC9513192; doi:10.3389/fendo.2022.960274)
Supplement: Supplementary file 4 [file Table_4.docx]

**Supplementary Table** **4.** Results of ROC analyses for distinguishing between PCOS women with and without HA

| **Lipid** | **AUC (95% CI)** | ***P* value** |
| --- | --- | --- |
| FFA C16:0 | 0.786 (0.608, 0.963) | 0.016 |
| FFA C18:1 | 0.903 (0.787, 1.000) | 0.001 |
| FFA C18:3 | 0.773 (0.578, 0.968) | 0.021 |
